# Bronchus‐Associated Lymphoid Tissue in Humans—The Past and Recent Research

**DOI:** 10.1111/imm.70025

**Published:** 2025-08-03

**Authors:** Thomas Tschernig, Reinhard Pabst

**Affiliations:** ^1^ Institute of Anatomy and Cell Biology Saarland University Homburg/Saar Germany; ^2^ Department of Immunomorphology, Centre of Anatomy Hannover Medical School Hannover Germany

A paper of Matsumoto and colleagues [1] is highly relevant for mucosal immunology as it presents new data of bronchus‐associated lymphoid tissue (BALT) in humans. In 1972, John Bienenstock and colleagues [2] described the aggregation of lymphoid tissue in the bronchial wall and introduced the term BALT. Those investigations were restricted to rabbits but soon this concept was included in many textbooks and BALT was described as a secondary lymphoid tissue comparable to Peyer's patches [3]. Nevertheless, in the lungs of normal human adults BALT was absent [4]. In contrast, BALT was detected in 8% of chronically inflamed human lungs [5]. In young children who died of SIDS or other reasons BALT was documented in about 40% [6]. In 2024, we documented that also in rabbits, BALT is dependent on environmental stimuli [7]. Later, inducible BALT (iBALT) was described by a couple of groups [8, 9, 10]. These structures seem to fulfil the criteria for a tertiary lymphoid tissue. However, not all of the reported follicular aggregations of lymphoid cells were located in the bronchial wall and then the term bronchus‐associated was not applicable. The recent data of Matsumoto et al. [11] support old findings of arising and dissolving of BALT in humans and support our two‐step concept: Stimulation by external stimuli to re‐induce BALT and then inhalation of vaccines to produce protective antibodies.

## Conflicts of Interest

The authors declare no conflicts of interest.

## Data Availability

Data sharing not applicable to this article as no datasets were generated or analysed during the current study.
